# Nasal Oxytocin Treatment Biases Dogs’ Visual Attention and Emotional Response toward Positive Human Facial Expressions

**DOI:** 10.3389/fpsyg.2017.01854

**Published:** 2017-10-17

**Authors:** Sanni Somppi, Heini Törnqvist, József Topál, Aija Koskela, Laura Hänninen, Christina M. Krause, Outi Vainio

**Affiliations:** ^1^Department of Equine and Small Animal Medicine, Faculty of Veterinary Medicine, University of Helsinki, Helsinki, Finland; ^2^Institute of Cognitive Neuroscience and Psychology, Hungarian Academy of Sciences, Budapest, Hungary; ^3^Department of Production Animal Medicine, University of Helsinki, Helsinki, Finland

**Keywords:** domestic dog, nasal oxytocin, facial expressions, eye movements, pupil diameter, emotional arousal

## Abstract

The neuropeptide oxytocin plays a critical role in social behavior and emotion regulation in mammals. The aim of this study was to explore how nasal oxytocin administration affects gazing behavior during emotional perception in domestic dogs. Looking patterns of dogs, as a measure of voluntary attention, were recorded during the viewing of human facial expression photographs. The pupil diameters of dogs were also measured as a physiological index of emotional arousal. In a placebo-controlled within-subjects experimental design, 43 dogs, after having received either oxytocin or placebo (saline) nasal spray treatment, were presented with pictures of unfamiliar male human faces displaying either a happy or an angry expression. We found that, depending on the facial expression, the dogs’ gaze patterns were affected selectively by oxytocin treatment. After receiving oxytocin, dogs fixated less often on the eye regions of angry faces and revisited (glanced back at) more often the eye regions of smiling (happy) faces than after the placebo treatment. Furthermore, following the oxytocin treatment dogs fixated and revisited the eyes of happy faces significantly more often than the eyes of angry faces. The analysis of dogs’ pupil diameters during viewing of human facial expressions indicated that oxytocin may also have a modulatory effect on dogs’ emotional arousal. While subjects’ pupil sizes were significantly larger when viewing angry faces than happy faces in the control (placebo treatment) condition, oxytocin treatment not only eliminated this effect but caused an opposite pupil response. Overall, these findings suggest that nasal oxytocin administration selectively changes the allocation of attention and emotional arousal in domestic dogs. Oxytocin has the potential to decrease vigilance toward threatening social stimuli and increase the salience of positive social stimuli thus making eye gaze of friendly human faces more salient for dogs. Our study provides further support for the role of the oxytocinergic system in the social perception abilities of domestic dogs. We propose that oxytocin modulates fundamental emotional processing in dogs through a mechanism that may facilitate communication between humans and dogs.

## Introduction

The hypothalamic neuropeptide oxytocin plays a significant role in the regulation of a variety of social behaviors in both humans and other mammals. Oxytocin is known especially as an “affection hormone” facilitating parental care and pair-bonding, but it can also affect other social behaviors, such as social approach, trust, cooperation and empathy (reviewed in Campbell, 2010; Bartz et al., 2011; Romero et al., 2016; Shamay-Tsoory and Abu-Akel, 2016). The influence of oxytocin on complex social behavior is likely based on its modulating effects on rudimentary social processing, as the perception of social cues and encoding emotional information (Bartz et al., 2011; Ellenbogen et al., 2012). It has been proposed that this neuropeptide enhances the salience of cues important for social interaction and can additionally reduce withdrawal behaviors in the presence of socially aversive cues (Bartz et al., 2011; Shamay-Tsoory and Abu-Akel, 2016). To understand more broadly how the oxytocinergic system mediates pro-social behavior and emotions, it is necessary to investigate the effects of oxytocin on basic social perception in different species (Parr et al., 2013; Kovács et al., 2016a).

Domestic dogs (*Canis familiaris*) may be ideal subjects for studying the behavioral phenomena related to oxytocin because they resemble humans more than any other animal with regard to those aspects of social-cognitive functioning (e.g., attachment to their human partners – Topál et al., 2009), which have been linked to the central release of oxytocin. For example, endogenous oxytocin levels rise both in humans and dogs after positive human–dog interactions (Odendaal and Meintjes, 2003; Miller et al., 2009; Nagasawa et al., 2009, 2015; Handlin et al., 2011, 2012; Mitsui et al., 2011; Rehn et al., 2014) and correlate with the quality of dog–owner relationship (Handlin et al., 2012). Interestingly, polymorphisms in the oxytocin receptor gene are related to contact seeking and amicability toward humans (Kis et al., 2014) suggesting that oxytocin may contribute to the genetic background of socio-cognitive behavior in dogs (Bence et al., 2013). The oxytocin-facilitated communication may have supported the development of interspecies relations between humans and dogs through the domestication of dogs (MacLean and Hare, 2015; Nagasawa et al., 2015).

Increasing evidence suggests that the intranasal administration of oxytocin underpins a valid approach to study mechanisms underlying social behavior and cognition in dogs (Kis et al., 2017). The inhaled oxytocin is able to cross the blood–brain barrier and thus has the potential to modulate the behavior of dogs by directly affecting the brain (Romero et al., 2014). Importantly, however, oxytocin probably exerts its effects through multiple mechanisms, such as enhancing social motivation, increasing the salience of social cues and reducing social anxiety (Bartz et al., 2011). Intranasal oxytocin treatment has been found to promote dogs’ affiliation toward both humans and conspecifics (Romero et al., 2014), to facilitate playful interactions between dogs (Romero et al., 2015), to increase eye contact in dog–human interactions (Nagasawa et al., 2015; Oliva et al., 2015; Kovács et al., 2016b) and to improve the reading of human body language (Oliva et al., 2015). Oxytocin biases dogs’ behavior toward positive expectations (Kis et al., 2015) and moderates dogs’ behavior both in affiliative (Romero et al., 2014; Nagasawa et al., 2015) and threatening interactions (Hernádi et al., 2015; Kovács et al., 2016b). The investigations, so far, have focused mainly on how oxytocin affects the dog’s behavior. However, it is likely that oxytocin regulates dogs’ socio-cognitive functions already at the level of visual perception (Kovács et al., 2016a).

In primates, oxytocin alters the interconnection patterns of those brain regions involved in attention, perception and emotion regulation (Kirsch et al., 2005; Gamer et al., 2010). Intranasal oxytocin administration improves the recognition and memorization of facial emotional expressions (e.g., Savaskan et al., 2008; Lischke et al., 2012, for a review see Shahrestani et al., 2013) and increases gazing at faces and eyes (in humans: Guastella et al., 2008a; Domes et al., 2012; in monkeys: Ebitz et al., 2013; Dal Monte et al., 2014). Typically, intranasal oxytocin suppresses vigilance to threatening social cues (in humans: Kirsch et al., 2005; Di Simplicio et al., 2009; Kim et al., 2014; in monkeys: Ebitz et al., 2013; Parr et al., 2013) but also improves visual processing of socially rewarding stimuli such as smiling faces (Guastella et al., 2008b; Domes et al., 2012, 2013). Dogs, to some extent, view and process faces like primates (Guo et al., 2009; Törnqvist et al., 2013; Somppi et al., 2014, 2016; Dilks et al., 2015; Cuaya et al., 2016). Dogs can distinguish human facial expressions from pictures and modulate their reactions in accordance with the emotional information in them (Nagasawa et al., 2011; Racca et al., 2012; Albuquerque et al., 2016; Barber et al., 2016; Somppi et al., 2016). However, there are no studies to date explicitly focusing on the effects of intranasal oxytocin on face perception in dogs.

Recent evidence suggests that during visual inspection of emotionally arousing events, pupils dilate in response to sympathetic nervous system activation (e.g., Bradley et al., 2008). Hence, pupil diameter can be used as a physiological index of emotional arousal (see Laeng et al., 2012 for a review). Oxytocin can promote social information gathering by affecting subjective emotional states and modulating pupil dilation (Ebitz et al., 2013; Leknes et al., 2013; Prehn et al., 2013) because the oxytocinergic system interacts with brain mechanisms regulating motivation, emotional arousal and attentional processes (Shamay-Tsoory and Abu-Akel, 2016). Examination of pupil dilation, therefore, may give a deeper perspective for the interpretation of gazing behavior.

It is increasingly accepted that oxytocin selectively amplifies social approach and inhibits social vigilance in both humans and non-human animals (e.g., Guastella et al., 2008a; Domes et al., 2012; Ebitz et al., 2013; Theodoridou et al., 2013; Romero et al., 2016), and in this study, we aimed to assess the validity of this assumption in the case of domestic dogs. In a placebo-controlled, within-subjects design, dogs after having received either oxytocin or a placebo (saline) treatment were presented with pictures of unfamiliar male human faces displaying either positive or negative emotional expressions. The voluntary attention (eye gaze patterns) and the indicator of sympathetic arousal state (changes in pupil dilation) were recorded by an eye tracking device. We hypothesized that intranasal oxytocin would affect the rudimentary attentional and affective processes in dogs which manifests itself in: (i) increased gaze toward the eye region of human faces, especially during the viewing of positive (smiling) faces (ii) decreased vigilance when presented with threatening facial expressions and, (iii) changes in emotional arousal as reflected in changes in pupil diameter.

## Materials and Methods

The experiments were conducted at the Veterinary Faculty of the University of Helsinki from August to October 2012. The oxytocin treatment was licensed by the Finnish Medicines Agency, Fimea (vetkl-nro05/2012). The procedures were approved by the Ethical Committee for the Use of Animals in experiments at the University of Helsinki (minutes 9/2012) and all dog owners completed an informed written consent to participate in the study.

### Animals and Pre-training

A total of 46 dogs were recruited for the study, but three dogs were not able to complete the final tests (because of illness, *n* = 1; nervousness, *n* = 1; unsuccessful calibration, *n* = 1). The final experimental group included eight 6-years-old purpose bred beagles housed in a group kennel (6 castrated males, 2 sterilized females) and 35 privately owned 1- to 10-years-old pet dogs (mean ± *SD*: 6.5 ± 2.2 years; 23 intact females, 7 sterilized females, 3 intact males and 2 castrated males). Pet dogs represented 16 different breeds and mongrels (**Table 1**). The average weight of the dogs was 20.5 kg (*SD* = 9.1, range: 3.5–40 kg; **Table 1**). None of the dogs were on medication during the study and the female dogs were in the anestrus phase. Throughout the whole study period, the daily routines of the dogs were kept similar to that in their regular life. Pet dogs were fed one to two times and taken outdoors three to five times daily. Kennel dogs lived in the kennel facilities at the University of Helsinki. They were fed twice a day, and once a day they were released into an exercise enclosure for 2 h.

**Table 1:** Breeds, ages, sex and weights of the subjects.

Breed	Age (years)	Sex	Weight (kg)	Group
Australian kelpie	6	Sterilized female	14	Pet dog
Beauce Shepherd	3.5	Female	38	Pet dog
Beauce Shepherd	4.5	Sterilized female	30	Pet dog
Beauce Shepherd	5	Female	36	Pet dog
Border collie	1	Female	15	Pet dog
Border collie	1.5	Male	20	Pet dog
Border collie	2.5	Female	19	Pet dog
Border collie	4	Female	13	Pet dog
Border collie	6.5	Female	15	Pet dog
Border collie	8	Castrated male	18	Pet dog
Border collie	10	Sterilized female	18.5	Pet dog
Bouvier	6.5	Female	30	Pet dog
Boxer	1.5	Female	27	Pet dog
Boxer	5.5	Female	26	Pet dog
German Shepherd	3	Female	27	Pet dog
German Shepherd	5	Female	30	Pet dog
German Shepherd	6	Female	27	Pet dog
Hovawart	3.5	Female	30	Pet dog
Hovawart	4.5	Sterilized female	27.5	Pet dog
Hovawart	7	Sterilized female	30	Pet dog
Lagotto Romagnolo	5	Male	15	Pet dog
Miniature schnauzer	3.5	Sterilized female	7	Pet dog
Mixed breed (half rottweiler)	2.5	Female	30	Pet dog
Mixed breed (half Labrador)	3.5	Female	32	Pet dog
Mixed breed (unknown breeds)	8.5	Sterilized female	20	Pet dog
Rottweiler	2.5	Female	40	Pet dog
Rough Collie	3.5	Female	21	Pet dog
Rough Collie	4	Female	20	Pet dog
Smooth collie	8	Male	24.5	Pet dog
Swedish Vallhund	6.5	Female	11	Pet dog
Toy poodle	5.5	Female	3.5	Pet dog
Toy poodle	2	Female	4.5	Pet dog
Welsh corgi cardigan	5	Female	16	Pet dog
Welsh corgi cardigan	6.5	Female	15	Pet dog
Welsh corgi cardigan	8	Castrated male	18	Pet dog
Beagle	6	Castrated male	12	Kennel dog
Beagle	6	Castrated male	11.5	Kennel dog
Beagle	6	Castrated male	16.5	Kennel dog
Beagle	6	Castrated male	12	kennel dog
Beagle	6	Castrated male	11.5	Kennel dog
Beagle	6	Castrated male	14.5	Kennel dog
Beagle	6	Sterilized female	12	Kennel dog
Beagle	6	Sterilized female	16	Kennel dog


Prior to conducting the experiments, the dogs were clicker trained to lie still and lean their chins on a specially designed rack, as described in Somppi et al. (2012). The criterion for passing the training period was that the dogs took the pre-trained position without being commanded to do so and remained in that position for at least 30 s while their owners and the experimenters were positioned behind an opaque barrier. During the training, the dogs were not encouraged to fix their eyes on a monitor or images and they were not restrained or forced to perform the task. Pet dogs were trained by their owners and kennel dogs were trained by the first and second authors. The owners with their dogs visited the experimental room approximately 2–3 (range 1–10) times prior to the actual experiment until they were fully familiar with the experimental environment and instrumentation setup. Most of the dogs visited the experimental room 2–3 times, but some dogs needed more visits because they had difficulties concentrating on the task.

### Eye Tracking System

The binocular eye movements of the dogs were recorded with an infrared-based contact free eye tracking system (iView X^TM^ RED250, SensoMotoric Instruments GmbH, Germany) which was integrated below a 22” LCD-monitor (1680 pixels × 1050 pixels) placed at 0.50–0.75 m (mean ± *SD*: 0.69 ± 0.05 m) distance from the dogs’ eyes.

During the tests, the chin rest, the monitor and the eye tracker were placed in a cabin constructed of plywood and cardboard. The cabin had one open wall, three solid walls, and a roof (*h* = 1.5 m, *w* = 0.9 m, *d* = 0.9 m). Two additional fluorescent lamps were placed in front and above the monitor. The average illumination intensity measured on the sides of the dogs’ heads was 11000 (*SD* = 2300 lx: range 4200–13400 lx). These differences in the photometric values (luminous intensity) were due to the fact that different sized dogs were placed at different distances from the monitor and light sources.

The eye tracker was calibrated and the calibration accuracy was checked twice using a five-point procedure (Somppi et al., 2012, 2014). The calibrated area was a visual field of 40.5° × 24.4° from the average distance of 0.70 m (equal to the size of the monitor). The criterion for an adequate calibration was achieved if the dogs’ eye fixations hit within a 1° radius off the central calibration point and at least three of four distal points. The fixation was encoded if the minimum fixation duration was 75 ms and the maximum dispersion value *D* = 250 pixels (Somppi et al., 2012). On average, five calibration trials were required for each dog to achieve an adequate calibration (*SD*: 4.2, range: 1–27). For the final calibrations, the average accuracy was 96% (*SD*: 8%, range: 70–100%), calculated as a proportion of fixated points out of five calibration points over two calibration checks of all dogs. To maintain vigilance and motivation of dogs, the calibration and image viewing were carried out on separate days. According to our previous findings, the stored calibration can be used repeatedly during separate days (Somppi et al., 2012). Illumination and the position of the chin rest, monitor, and eye tracker were kept the same during the calibration and the image viewing. The accuracy of the central point fixations was re-assessed visually immediately before the image viewing.

### Stimuli

**FIGURE 1:**
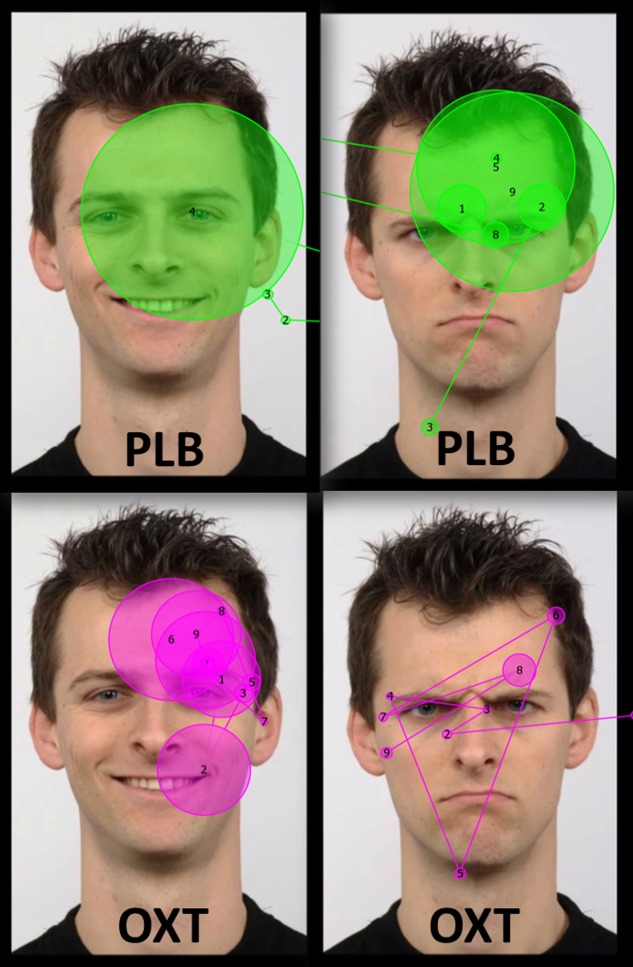
Examples of the stimuli images (from Radboud faces database; Langner et al., 2010) and scan paths of one dog during 7000 ms viewing after the placebo (PLB) and the oxytocin treatments (OXT). The dotted white rectangular area in the top left image represents the analyzed area of interest. Circles represent the gaze fixations of one dog and the lines trace the path that the gaze traveled across the image. Circle size is proportional to the fixation time. The numbers in the circles represent the order of the fixations.

A total of eight digital color facial photographs from the Radboud Faces Database (RaFD, Langner et al., 2010) were used (see an example in **Figure 1**). We selected four male faces with happy (smiling, teeth visible) and angry (teeth not visible) expressions. The size of the image area was an average 800 pixels × 580 pixels (height 800 pixels, width range 500–620 pixels depending on the width of the faces). The stimuli were presented on a black background using Experiment Center 3.0^TM^ software (SensoMotoric Instruments GmbH, Berlin, Germany).

During one testing session, one happy face and one angry face (‘a stimulus set’) of the same male person, unfamiliar for the participating dogs, were presented consecutively on the left or on the right side of the screen in altering orders, 7 s per image. During the face presentation, a 3-s-long neutral sound is played. Before each face presentation a 4-s-long ‘attractor,’ a swinging pendulum and tick-tock sound, was presented to the dogs in order to get the dogs’ attention. The stimulus sets were pseudo-randomized between the dogs.

### Experimental Procedure

In a balanced within-subject experimental setup the dogs were given oxytocin nasal spray (OXT; Syntocinon^®^ 40 IU/ml, Novartis, Australia) in one testing session and a placebo saline nasal spray (PLB; Naso NaCl 0.9%, Ratiopharm, Germany) in other testing session, 7–25 days (mean interval ± *SD*: 9.7 ± 4 days) between them. Different stimulus sets were presented in the first and second testing sessions.

The experimental setup consisted of four phases. First, the dogs were brought to the test room for warm-up trials, in which they viewed a series of images of landscapes and wild animals while the experimenter rewarded them randomly after one to five images. The warm-up phase lasted for 5–10 min, depending on the dog’s behavior. After the warm-up trials, the dogs were intranasally administered 12 IU PLB or OXT in a different room (3 puffs in alternating nostrils; the sequence for half of the dogs was right-left-right while for the others left-right-left). Only two puffs were given (one in each nostril) for dogs that weighed less than 5 kg. After the treatment, subjects waited in their owners’ car or in a separate room without social stimulation. Dog owners were instructed to avoid social contact with their dogs and to avoid getting the dog excited (i.e., no stroking, playing or training during the waiting time). Finally, the dog was brought back into the testing room. It settled down in the pre-trained position, and eye tracking data recording was started after the eye tracker had detected the dog’s eye properly. The dog was rewarded after the stimulus presentation regardless of its behavior. If the dog changed its predetermined position during the stimulus presentation (i.e., lifted its head from the chin rest), it was not re-positioned by the experimenter/owner. The average time that elapsed between the nasal spray administration and the presentation of stimulus set was 46 min (*SD*: 2 min, range 44–61 min).

The data from original testing session of six dogs were lost due to eye tracker software crashes (*n* = 2) or the dogs lifting their heads from the chin rest (*n* = 4). These testing sessions were redone 23–33 days later with the same treatment as in the original test (2 dogs PLB, 4 dogs OXT), but with a different stimulus set.

### Data Analysis

Eye movement data were obtained from a total of 169 images (PLB Happy *n* = 43; PLB Angry *n* = 40; OXT Angry *n* = 43; OXT Happy *n* = 43). Data from three dogs in the PLB/Angry image condition were insufficient for analysis because of technical problems or due to dogs’ head movements.

Different aspects of dogs’ gazing behavior toward a rectangular eye region (area of interest – see **Figure 1**), which was one-third the size of the entire face region, were recorded. Two gaze variables derived from eye movement data were considered for analyses: fixation count (the sum of all fixations that hit on the eye region during the entire stimulus presentation time) and revisits (how many times the dog glances back at the eye region). In addition, the average pupil size (pupil diameter in mm; recorded during the fixations targeted at the monitor) was calculated using the manufacturer’s built-in algorithm. (BeGaze 3.0^TM^ software, SensoMotoric Instruments GmbH, Berlin, Germany).

The effects of the treatment (OXT or PLB) on the gazing behavior (number of fixations, revisits) and pupil diameter were analyzed with generalized linear mixed models (GENLINMIXED, SPSS 24.0, IBM, New York, NY, United States) using normal distribution and identity link function with first-order autoregressive covariance structure (AR1). The model fitting was based on the evaluation of Akaike Information Criteria and Pearson residual observed-by-predicted plots. The fixed factors included in the final model were treatments (OXT or PLB), facial expression (Happy or Angry) and the interaction between the facial expression and the treatment. The subject (i.e., tested dog) and interaction between the treatment and the testing day were included as random effects, latter to take into account the cross-over design of the experiment. The testing day and sequence of the image were included as repeated measures. In the analysis for the pupil diameter, total number of fixations was used as a covariate to take into account the possible dependency between gaze variables and pupil dilation (Aspinall et al., 2014; Ebitz et al., 2014). The sex and dog population (pet or kennel dog) were tested both as fixed and random effects, but discarded from the final analysis because they were statistically significant (*p* > 0.05) as fixed effects, and redundant for the models as random effects. The *post hoc* tests with sequential Bonferroni adjustment were included in the GENLINMIXED procedure. The gazing variables were log-transformed for analyzes to acquire better model fitting, and they are reported as log-transformed values. All results are reported as estimated means with their standard errors (SE) using the significance level *p* < 0.05.

## Results

### Number of Fixations

**FIGURE 2:**
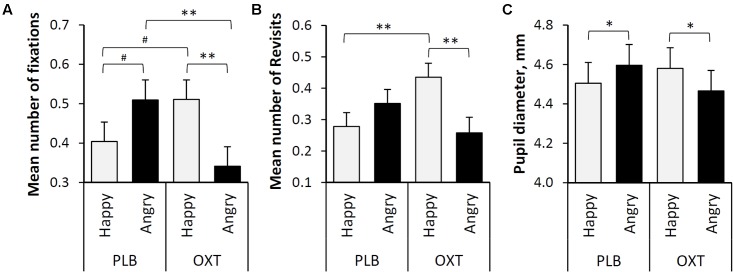
**(A)** The mean number of fixations targeted to the eye regions of happy and angry faces (Lg10 transformed values). **(B)** The mean number of revisits targeted to the eye regions of happy and angry faces (Lg10 transformed values). **(C)** The mean pupil diameter (mm) during the presentation of two facial expressions (Happy and Angry) and two treatments (PLB and OXT). Error bars represent *SE*. Asterisks indicate significant differences (^∗∗^*p* < 0.01, ^∗^*p* < 0.05) and a statistical trend (^#^*p* < 0.09).

The overall mean number of fixations on the eye region of the human faces did not differ significantly between oxytocin and placebo treatments (*F* = 0.499, *df* = 165, *p* = 0.481) or between happy and angry facial expressions (*F* = 0.555, *df* = 165, *p* = 0.457). However, the interaction between the treatment (OXT/PLB) and facial expressions (Happy/Angry) was statistically significant (*F* = 10.182, *df* = 165, *p* = 0.002). *Post hoc* tests revealed that dogs fixated at the eye region of angry faces less frequently after oxytocin than after the placebo treatment (*t* = -2.721, *df* = 165, *p* = 0.007, **Figure 2A**) and tend to fixate at the eye region of happy faces more after oxytocin than after the placebo treatment, although this was not significant (*t* = 1.767, *df* = 165, *p* = 0.079, **Figure 2A**). After the placebo treatment dogs tended to fixate at the eyes of the angry faces more than eyes of the happy faces, although this was not significant (*t* = 1.711, *df* = 165, *p* = 0.089, **Figure 2A**). Oxytocin treatment instead induced more fixations toward the eye region of happy faces compared to the corresponding region of angry faces (*t* = 2.813, *df* = 165, *p* = 0.006, **Figure 2A**).

### Revisits

The overall mean number of revisits made to the eye regions of the human faces did not differ significantly between oxytocin and placebo treatments (*F* = 0.657, *df* = 116, *p* = 0.419) or between happy and angry expressions (*F* = 1.560, *df* = 116, *p* = 0.214). However, the interaction between the treatment (OXT/PLB) and facial expressions (Happy/Angry) was statistically significant (*F* = 8.908, *df* = 116, *p* = 0.003). *Post hoc* tests revealed that dogs revisited on the eye regions of happy faces more frequently (i.e., glanced back to eyes) after oxytocin treatment than after placebo treatment (*t* = 2.854, *df* = 116, *p* = 0.005). In addition, after the intranasal administration of oxytocin, the eye regions of happy faces were revisited more often than those of the angry faces (*t* = 2.909, *df* = 116, *p* = 0.004, **Figure 2B**).

### Pupil Diameter

Similarly, to ‘Number of fixations’ and ‘Revisits’, the overall mean pupil diameter of dogs while viewing human emotional faces did not differ significantly between oxytocin and placebo treatments (*F* = 0.107, *df* = 163, *P* = 0.744) or between happy and angry expressions (*F* = 0.142, *df* = 163, *p* = 0.706). However, the interaction between treatment (OXT/PLB) and facial expressions (Happy/Angry) was statistically significant (*F* = 9.998, *df* = 163, *p* = 0.002). *Post hoc* pairwise comparisons revealed that after the placebo treatment dogs had larger pupils while viewing angry as compared to happy faces (*t* = 2.488, *df* = 163, *p* = 0.014), but oxytocin treatment caused the opposite pupil response (*t* = -2.95, *df* = 163, *p* = 0.038, **Figure 2C**).

## Discussion

Intranasal administration of oxytocin modulates different aspects of social cognitive functioning in social mammals, including humans (Campbell, 2010; Romero et al., 2016). The behavioral effects of oxytocin are also increasingly evidenced in domestic dogs (Romero et al., 2014; Hernádi et al., 2015; Kis et al., 2015; Nagasawa et al., 2015; Oliva et al., 2015), an important model animal for studying the evolutionary emergence of human social cognition (Topál et al., 2009). Based on these results, the purpose of the present study was twofold: (i) we explored how oxytocin influences the allocation of visual attention in dogs when viewing emotionally expressive human faces and, (ii) we also investigated, for the first time in domestic dogs, the effects of oxytocin treatment on pupil size modulations related to attention allocation.

The results show that intranasal oxytocin treatment has a significant effect on both attentional focus and emotional arousal confirming that neuropeptide oxytocin mediates social perception and emotional states in dogs. Intranasal administration of oxytocin selectively attenuated dogs’ attention to the eye region of angry faces and reduced emotional arousal, reflected in their pupil sizes, when viewing negative facial expressions. In contrast oxytocin increased both emotional arousal and the allocation of attention toward the eye region of emotionally positive (smiling) faces. Thus, oxytocin treated dogs made fewer fixations on the eyes of angry faces and more revisits to the eyes of happy faces compared with the placebo treatment. Moreover, after oxytocin treatment dogs fixated and revisited more frequently the eye region of happy than angry faces. However, oxytocin did not increase the dogs’ gazing toward human eyes at general level, which is in line with the results of human and monkey studies showing that oxytocin administration does not generally enhance approaching social stimuli of any kind, but specifically increases the salience of particular social stimuli (Domes et al., 2012; Theodoridou et al., 2013; Dal Monte et al., 2014).

It seems that oxytocin treatment has the potential to bias dogs’ attention away from threat and toward smiling faces due the social anxiety relieving and pro-social behavior promoting properties of the neurohormone. This is in line with earlier reports suggesting that oxytocin promotes social interactions via focusing attention on social signals of potentially approaching friendly encounters and reducing social contact with potentially threatening encounters (Domes et al., 2012). Dogs, like many other animals, may consider direct eye contact threatening, even in artificial contexts (Somppi et al., 2016). Threatening stimuli typically evokes prolonged attention, which is the consequence of delayed disengagement of attention from threat (Fox et al., 2002; Belopolsky et al., 2011). The fear-attenuating effect of oxytocin, however, may reduce this response and allows more flexible processing of positive stimuli (Kirsch et al., 2005; Guastella et al., 2008a,b; Bartz et al., 2011; Ebitz et al., 2013; Parr et al., 2013; Theodoridou et al., 2013; Kim et al., 2014). Furthermore, oxytocin increases trustworthy and approach behavior (Bartz et al., 2011; Romero et al., 2014, 2016; Shamay-Tsoory and Abu-Akel, 2016), which may have increased the dogs’ willingness to look at the smiling eyes as well.

Previous studies have shown that oxytocin increases attention to the eye region of conspecific faces (Guastella et al., 2008a; Andari et al., 2010; Ebitz et al., 2013; Dal Monte et al., 2014), in domestic dogs, however, this phenomenon seems to go beyond the boundaries between species. The present study provides the first evidence that oxytocin can affect the visual processing of heterospecific emotional facial expressions, which is consistent with the observations showing that exogenous oxytocin promotes dogs’ social behaviors toward human partners. Oxytocin facilitates interspecies attachment by enhancing dogs’ motivation to approach and affiliate with humans (Romero et al., 2014; Nagasawa et al., 2015). Recently, Nagasawa et al. (2015) have found that after intranasal oxytocin administration dogs gazed more toward their owners’ faces, which consequently facilitated owners’ affiliative behavior toward their dogs, suggesting that the mutual oxytocin-mediated gaze between dog and owner promotes human–dog-bonding. Oxytocin also improves dogs’ ability to interpret the social cues of humans other than their owners, probably because oxytocin helps dogs to tolerate shifting their gaze to the human eyes (Oliva et al., 2015; Kovács et al., 2016b). Oxytocin increased looking back at the eyes of friendly faces also in our artificial setup suggesting that oxytocin has a role in maintaining prolonged eye contact at a very rudimentary level.

The enhanced eye gazing during viewing of emotional faces is likely related to dogs’ emotional state. As an indicator of emotional arousal (Bradley et al., 2008; Machado et al., 2011), we measured the pupil diameters of the dogs and found that oxytocin-treated dogs had larger pupils while viewing happy faces while placebo-treated dogs had larger pupils while viewing angry faces. In humans, greater pupil dilation is associated with both visual attention and increased emotional arousal (Bradley et al., 2008; Gredebäck et al., 2012; Kret et al., 2013). It is noteworthy that oxytocin can both increase positive arousal and attenuate negative arousal related to fear reactions (Bradley et al., 2008; Kret et al., 2013; Ellenbogen et al., 2014) and pupils can dilate due to either pleasant or unpleasant emotional states (Bradley et al., 2008; van Steenbergen et al., 2011), as we found in dogs.

Altogether the link between gazing patterns and pupil dilation in dogs provides further support for the notion that fluctuations in pupil diameter may reflect allocation of attentional resources (Leknes et al., 2013). Oxytocin can directly affect the effectiveness of social information gathering by selectively modulating pupil dilation (Leknes et al., 2013; Prehn et al., 2013; Ebitz et al., 2014). Pupil dilation-linked arousal may adjust the balance of processing resources in those situations in which both goal-relevant stimuli and conflicting but biologically important objects compete for recruiting the subject’s attention (Ebitz et al., 2014). Attention is typically focused on stimuli of great biological and social significance, such as threatening stimuli if a subject is in a negative arousal state. Oxytocin may control pupil dilation through attenuation of negative arousal thus biasing attention toward other relevant targets (Prehn et al., 2013; Ebitz et al., 2014). Although the happy and angry faces in our study were not actually competing for the dogs’ attention (i.e., stimuli were presented consecutively), the changes in the dogs’ focus of attention and pupil dilation were probably based on the aforementioned mechanism. According to a recent theoretical framework, oxytocin has a major role in regulation of social attention through its interaction with the dopaminergic system and amygdala, both brain regions responsible for emotional arousal, rewarding system and detection of socially relevant stimuli (Shamay-Tsoory and Abu-Akel, 2016). We suggest that after receiving placebo treatment angry faces were more relevant and salient for dogs because seeing the directly staring threatening face induced negative emotional arousal in them. Conversely, oxytocin treatment reduced this social anxiety response and probably also facilitated the positive arousal evoked by the viewing of smiling faces. It should be noted that one factor which may have affected the results is the general emotional state of dogs, which was positive as dogs were highly motivated to participate in the task due to reward based positive operant conditioning. Importantly, however, eye movements and pupil data alone are insufficient to draw firm conclusions about dog’s emotional state or oxytocin’s role in attention and emotion regulation in dogs.

Further studies are needed to determine whether the results we report are typical only for domestic dogs due their human-tunedness in their communicative skills, or, whether a similar phenomenon exists in other mammalian species. Moreover, in our study dogs were presented with unfamiliar human faces, thus, at present we do not know whether familiarity would modulate the observed effect of oxytocin on emotional face processing. Recent findings highlight the potential role of familiarity: familiar faces attract dogs’ attention more than unfamiliar ones (Somppi et al., 2014) and the effect of oxytocin may be more pronounced toward socially more relevant partners (Hernádi et al., 2015). In further studies, therefore, both stimuli of familiar/unfamiliar conspecifics, hetero-specifics as well as inanimate object should also be tested to clarify the effects of oxytocin on dogs’ gazing behavior. We may assume that oxytocin regulates the viewing of own-species faces differently, because the effects of oxytocin are highly context dependent (Shamay-Tsoory and Abu-Akel, 2016). Besides, the emotional signals of other-species and own-species may elicit differential viewing strategies in dogs (Somppi et al., 2016).

In future studies, the effects of the dogs’ breed, sex and personality traits should also be taken into account, as the impact of oxytocin are not uniform across all individuals (Kovács et al., 2016b; Shamay-Tsoory and Abu-Akel, 2016). Previous studies had also reported both sex- and breed-specific effects of intranasal oxytocin: e.g., female dogs and dog breeds selected for enhanced cooperative abilities were found to be more susceptible to the effects of intranasal oxytocin (Kis et al., 2014, see also Nagasawa et al., 2015; Kovács et al., 2016a,b). The subjects of our eye tracking experiment, however, were not appropriate for investigating sex and breed effects: the vast majority of the dogs were females (32/43) and most of the participant dogs represent herding dog breeds (22/43),other breeds selected for cooperative work (11/43, including two mixed breed dogs with known pedigree). We should note, however, that a potential confound to the effect of oxytocin in this (and many previous) research is that breed and sex as well as age, reproductive viability, size, pre-training success and time between testing sessions varied among subjects. Further studies are needed to examine whether these differences have a real impact on the oxytocin-mediated effects of dogs’ facial emotion processing or not.

## Conclusion

Both gazing patterns and pupillary data underpin the links between oxytocinergic, attentional and emotional circuits in domestic dogs. Oxytocin administration selectively biases the emotional arousal and attention allocation in domestic dogs by suppressing vigilance toward threatening social stimuli and increasing the arousal inducing effect of smiling human faces and making the eye gaze of friendly humans more salient for dogs. Taken together, our study support the hypothesis that oxytocin modulates fundamental emotional processing in dogs through a mechanism that facilitates communication between humans and dogs.

## Author Contributions

Conceptualization: JT, SS, and HT. Methodology: SS, HT, JT, AK, and CK. Data collection: AK, SS, and HT. Data analyses: SS, LH, and HT. Contributed in writing: SS. Contributed in review and editing: LH, HT, OV, AK, CK, and JT.

## Conflict of Interest Statement

The authors declare that the research was conducted in the absence of any commercial or financial relationships that could be construed as a potential conflict of interest.
